# The association between aspirin use and immune-related adverse events in specific cancer patients receiving ICIs therapy: analysis of the FAERS database

**DOI:** 10.3389/fphar.2023.1259628

**Published:** 2023-11-15

**Authors:** Huaju Yang, Zheran Liu, Ruidan Li, Rendong Huang, Xingchen Peng

**Affiliations:** ^1^ Department of Radiation Oncology, Cancer Center, West China Hospital, Sichuan University, Chengdu, China; ^2^ Department of Biotherapy, Cancer Center, West China Hospital, Sichuan University, Chengdu, China; ^3^ Hangzhou Linan Guorui Health Industry Investment Co., Ltd., Hangzhou, China

**Keywords:** immune checkpoint Inhibitors (ICIs), aspirin, immune-related adverse events (irAEs), cancer, US food and drug administration adverse event reporting system (FAERS) database

## Abstract

**Background:** The promise of immune checkpoint inhibitors (ICIs) therapy in cancer treatment is tempered by the occurrence of immune-related adverse events (irAEs). Many patients undergoing ICIs also take aspirin, but the association between aspirin and irAEs is not well understood.

**Methods:** This study analyzed adverse reaction data associated with the use of ICIs in the US Food and Drug Administration (FDA) Adverse Event Reporting System FDA Adverse Event Reporting System database, from the approval date of each drug until 1 October 2022. Multivariate logistic regression was employed to assess the association of aspirin use with irAEs in patients receiving ICIs.

**Results:** The results indicated that aspirin use was associated with an increased risk of irAEs in a pan-cancer analysis, with a more pronounced association in specific cancer types such as lung cancer, mesothelioma, and pancreatic cancer. However, in lymphoma, aspirin use was associated with a reduced risk of irAEs. Furthermore, aspirin use was associated with an increased risk of certain irAEs, such as anemia, colitis, myocarditis, myositis, pancreatitis, pericarditis, and pneumonia, while it was associated with a reduced risk of rash, Stevens-Johnson syndrome, and thyroiditis.

**Conclusion:** This study has unveiled an association between aspirin use and irAEs in cancer patients receiving ICIs therapy, emphasizing the need for individualized consideration of patients’ medication history when devising cancer treatment plans to enhance efficacy and reduce risks.

## 1 Introduction

ICIs therapy is a groundbreaking approach to treating tumors that leverages the immune system to combat malignancies. This approach enhances immune-mediated tumor clearance by blocking negative signals between cancer cells and immune cells (Waldman et al., 2020; Morad et al., 2021). To this end, ICIs that target programmed cell death-1 (PD-1)/programmed death ligand-1 (PD-L1) as well as cytotoxic T lymphocyte-associated protein 4 (CTLA-4) have been developed and employed by researchers in clinical practice (Waldman et al., 2020; Morad et al., 2021). As mounting evidence supports their efficacy and synergistic effects with other cancer treatments, ICIs are increasingly being utilized as a key component in the treatment of many types of cancer, such as melanoma, lung cancer and esophageal cancer (Gadgeel et al., 2020; Rudin et al., 2020; Doki et al., 2022; Livingstone et al., 2022). However, it is important to note that, to date, ICIs remains ineffective for several cancer types, for instance, pancreatic cancer (Bockorny et al., 2022). In some cases, ICIs have not yet attained the status of standard care, as seen in breast cancer (Debien et al., 2023).

However, a notable issue arising from the increasing use of ICIs in clinical practice is their uncontrolled additive impact on the immune system, resulting in irAEs. ICIs manifest unique patterns of toxicity distinct from conventional chemotherapy or other biological agents, often stemming from hyperactive immune reactions against normal organs. irAEs can affect any organ system, including the skin, gastrointestinal tract, cardiovascular system, and endocrine system, among others (Brahmer et al., 2018; Schneider et al., 2021). The frequency of irAEs ranged from 66.4% to 86.8% for all grades, and from 14.1% to 28.6% for grade 3 or higher (Xu et al., 2018). irAEs may be influenced by the patient’s genetic background and microbiome, as well as by treatment-related factors such as combination medication (Jelinic et al., 2018; Cortellini et al., 2020). The mechanism of irAEs is not fully understood but may be related to the overactivation of innate and adaptive immunity caused by the disruption of immune balance by immunotherapy (Pauken et al., 2019). Since the occurrence of irAEs restricts the use of ICIs, it is necessary to further understand the mechanism and influencing factors of irAEs.

Drug-drug interactions (DDI) are a significant focus in the field of systemic anti-cancer treatment. Previous studies have found that combination therapy has an important impact on the outcome of immunotherapy and irAEs. For example, the use of antibiotics and proton pump inhibitors (PPIs) has been associated with poorer outcomes in patients with ICIs (Kostine et al., 2021). Aspirin has become widely used in modern medicine, primarily due to its ability to inhibit the cyclooxygenase (COX) pathway and effectively treat inflammation, pain, and various cardiovascular diseases (Fijałkowski et al., 2022). In recent years, aspirin has also been found to have a well-documented role in the prevention and treatment of tumors (Algra and Rothwell, 2012; Rothwell et al., 2012), especially in colorectal cancer (Rothwell et al., 2010; Drew and Chan, 2021). With the innovation in the field of cancer treatment and the emergence of a new therapy, namely, immunotherapy, researchers have gradually paid attention to the relationship between aspirin and immunotherapy. Recent clinical studies have suggested that the combination of aspirin and ICIs is associated with better outcomes (Cortellini et al., 2020; Zhang et al., 2021). In addition, Aspirin use and its relationship to irAEs were rarely addressed in these studies. Given the widespread acceptance of ICIs into standard practice, it is crucial to gain a better understanding of the association between aspirin treatment and irAEs.

FAERS is a comprehensive drug adverse reaction database maintained by the FDA. Its advantages include broad coverage of adverse events from clinical trials to market use, timely updates, comprehensive drug information, large-scale data for analysis, and reliable reporting from healthcare professionals and consumers. It is a trusted resource for monitoring and reporting drug adverse reactions, and helps to inform better clinical practice and healthcare decision-making. To date, no systematic evaluation of the association of aspirin with irAEs has been published. Therefore, our aim was to determine the association between aspirin use and irAEs in patients receiving immunotherapy by analyzing the data in FAERS. Our research affirms that aspirin users exhibited a higher risk of irAEs when compared to non-aspirin users. Nonetheless, this association displayed variability across distinct cancer types, adverse events, and ICIs.

## 2 Methods

### 2.1 Data sources

The study utilized data from the FAERS database, a public repository that houses information on adverse events and medication errors reported to the FDA. This database is an essential tool for the FDA’s post-marketing safety surveillance program for drug and therapeutic biologic products. All data used for this analysis can be accessed at https://fis.fda.gov/sense/app/95239e26-e0be-42d9-a960-9a5f7f1c25ee/sheet/7a47a261-d58b-4203-a8aa-6d3021737452/state/analysis.

### 2.2 Data collection and screening

Adverse event (AE) reports from ICIs in the FAERS database were collected for this retrospective study. The analysis included every report from the date of each drug’s FDA approval until 1 October 2022. ICIs mainly consists of PD-1 inhibitors (Nivolumab, Pembrolizumab, Cemiplimab, Sintilimab, Camrelizumab, Tislelizumab, Toripalimab), PD-L1 inhibitors (Durvalumab, Atezolizumab, Avelumab), CTLA-4 inhibitors (Ipilimumab, Tremelimumab, Quavonlimab, Bms-986249), Lymphocyte-activation gene 3 (LAG-3) inhibitors (Opdualag, Relatlimab, Favezelimab, Fianlimab), PD-1/LAG-3 bispecific inhibitors (Nivolumab\Relatlimab-Rmbw, Tebotelimab). According to the patient’s medication, the treatment regimen were classified as monotherapy, dual immunotherapy, immunotherapy combined with targeted therapy, immunotherapy combined with chemotherapy, and immune combined antibody drug conjugates (ADC). We defined the use of aspirin during immunotherapy as aspirin users. irAEs were defined using AE terminology from the peer-reviewed immune-related adverse event (irAE) management guidelines (Martins et al., 2019). Patients with at least one irAE were categorized into the irAE group. The irAEs were sorted into primary system organ classes according to the Medical Dictionary for Regulatory Activities (Jing et al., 2022).

### 2.3 Statistical analysis

In this study, multivariable logistic regression was utilized to analyze adjusted odds ratios (OR) for evaluating the association between aspirin use and irAEs. The model included covariates such as age, sex, ICIs drugs, and treatment regimen. To account for multiple comparisons, Benjamini–Hochberg adjustment was performed using the “p.adjust” function in the “stats” R package. All comparisons are two tailed, and statistical significance was set at an FDR adjusted *p* < 0.05. The data were processed and analyzed using R statistical software version 4.2.1. On the overall population, we conducted multivariate regression analyses grouping by different tumor types, types of adverse reactions, and system organ classes (SOCs) to determine the impact of aspirin use on irAEs in patients treated with ICIs. Additionally, to further determine if different ICIs had an effect on the results, we performed multivariate logistic regression analyses in patients treated with PD-1 inhibitors, PD-L1 inhibitors, and CTLA-4 inhibitors, respectively.

## 3 Results

### 3.1 Baseline characteristics of patients

We collected information on 123,104 patients from FAERS and conducted a multivariate regression analysis (Table 1). Out of these patients, 70,655 were treated with PD-1 inhibitors, 21,315 were treated with PD-L1 inhibitors, 30,653 were treated with CTLA-4 inhibitors, 258 were treated with LAG-3 inhibitors, and 223 were treated with PD-1/LAG-3 bispecific inhibitors (Table 1). Moreover, 5,359 patients (4.4%) reported also taking aspirin (Table 1).

**TABLE 1:** Baseline feature.

Characteristics		With ASA	Without ASA	*p*-Value
		n = 5,359	n = 117,745	
**Sex**	Female	1,424 (26.6%)	41,676 (35.4%)	*p* < 0.001
	Male	3,909 (72.9%)	70,432 (59.8%)	
	Not specified	26 (0.5%)	5,637 (4.8%)	
**age**		68.9 (9.0)	63.9 (12.5)	*p* < 0.001
**ICIs type**	PD-1 inhibitor	2,847 (53.1%)	67,808 (57.6%)	*p* < 0.001
	PD-L1 inhibitor	1,193 (22.3%)	20,122 (17.1%)	
	CTLA-4 inhibitor	1,297 (24.2%)	29,356 (24.9%)	
	LAG-3 inhibitor	19 (0.6%)	239 (0.2%)	
	PD-1/LAG-3 inhibitor	3 (0.1%)	220 (0.2%)	
**Cancer type**	Bile duct cancer	15 (0.3%)	493 (0.4%)	*p* < 0.001
	Brain cancer	17 (0.3%)	641 (0.5%)	
	Breast cancer	57 (1.1%)	2,356 (2.0%)	
	Cervical cancer	8 (0.1%)	488 (0.4%)	
	Colorectal cancer	98 (1.8%)	1870 (1.6%)	
	Endometrial cancer	38 (0.7%)	1,227 (1.0%)	
	Esophageal cancer	48 (0.9%)	1,382 (1.2%)	
	Gastric cancer	52 (1.0%)	2,706 (2.3%)	
	Head and neck cancer	119 (2.2%)	2,996 (2.5%)	
	Liver cancer	159 (3.0%)	3,589 (3.0%)	
	Lung cancer	1,532 (28.6%)	29,212 (24.8%)	
	Lymphoma	81 (1.5%)	1732 (1.5%)	
	Melanoma	1,095 (20.4%)	25,904 (22.0%)	
	Mesothelioma	70 (1.3%)	1,256 (1.1%)	
	Metastatic tumor	112 (2.1%)	2,303 (2.0%)	
	Neuroendocrine tumor	13 (0.2%)	339 (0.3%)	
	Ovarian cancer	49 (0.9%)	1,212 (1.0%)	
	Pancreatic cancer	243 (4.5%)	1,691 (1.4%)	
	Prostate cancer	140 (2.6%)	1,025 (0.9%)	
	Renal cancer	591 (11.0%)	12,087 (10.3%)	
	Sarcoma	55 (1.0%)	843 (0.7%)	
	Skin cancer	39 (0.7%)	662 (0.6%)	
	Thyroid cancer	9 (0.2%)	299 (0.3%)	
	Urothelial tract cancer	165 (3.1%)	3,063 (2.6%)	
	Other cancers	554 (10.3%)	18,369 (15.6%)	
**irAEs**	Yes	1,294 (24.1%)	25,205 (21.4%)	*p* < 0.001
	No	4,065 (75.9%)	92,540 (78.6%)	

ASA: Aspirin.

### 3.2 Association of aspirin treatment with irAEs in different cancer types

**FIGURE 1:**
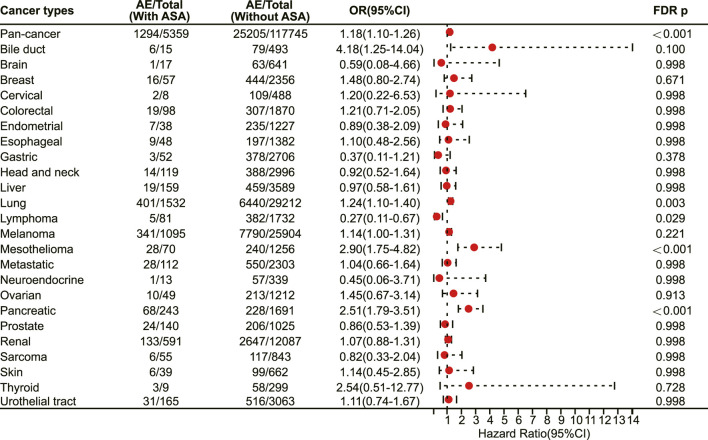
The forest plot showing the association between aspirin and irAEs in different cancer types among patients receiving immunotherapy. ASA: Aspirin.

The multivariate logistic regression analysis results revealed that aspirin use was associated with an increased risk of irAEs in the pan-cancer analysis (odds ratio (OR) 1.18, 95% confidence interval (CI) 1.10–1.26, FDR adjusted *p* < 0.001) (Figure 1). After excluding cancer types with a sample size of less than 200, we included 24 cancer types for analysis (Table 1). The further analysis indicated that aspirin use was linked to a higher risk of irAEs in specific cancer types. Specifically, aspirin use was significantly associated with an increased risk of irAEs in lung cancer (OR 1.24, 95% CI 1.10–1.40, FDR adjusted *p* = 0.003) (Figure 1), mesothelioma (OR 2.90, 95% CI 1.75–4.82, FDR adjusted *p* < 0.001) (Figure 1), and pancreatic cancer (OR 2.51, 95% CI 1.79–3.51, FDR adjusted *p* < 0.001) (Figure 1). In contrast, aspirin use was linked to a lower risk of irAEs in lymphoma (OR 0.27, 95% CI 0.11–0.67, FDR adjusted *p* = 0.029) (Figure 1). However, no significant differences in irAEs were observed in the remaining cancer types (Figure 1).

### 3.3 Association of aspirin treatment with different irAEs

**FIGURE 2:**
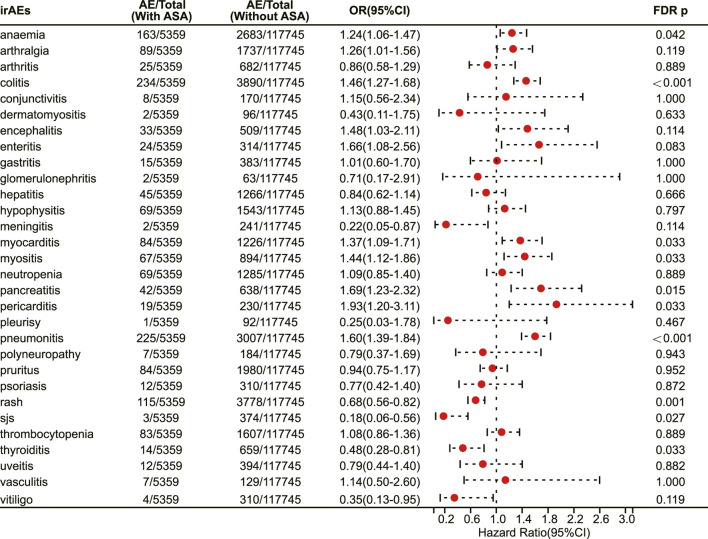
The forest plot showing the association between aspirin use and different irAEs among patients receiving immunotherapy. ASA: Aspirin; sjs: Stevens-Johnson syndrome.

We conducted a survey to determine the association of aspirin with specific irAEs. Our results revealed that aspirin use was correlated with an elevated risk of several adverse reactions, including anaemia (OR 1.24, 95% CI 1.06–1.47, FDR adjusted *p* = 0.042) (Figure 2), colitis (OR 1.46, 95% CI 1.27–1.66, FDR adjusted *p* < 0.001) (Figure 2), myocarditis (OR 1.37, 95% CI 1.09–1.71, FDR adjusted *p* = 0.033) (Figure 2), myositis (OR 1.44, 95% CI 1.12–1.86, FDR adjusted *p* = 0.033) (Figure 2), pancreatitis (OR 1.69, 95% CI 1.23–2.32, FDR adjusted *p* = 0.015) (Figure 2), pericarditis (OR 1.93, 95% CI 1.20–3.11, FDR adjusted *p* = 0.033) (Figure 2) and pneumonitis (OR 1.60, 95% CI 1.39–1.84, FDR adjusted *p* < 0.001) (Figure 2). On the other hand, aspirin use was associated with a decreased risk of certain adverse reactions, such as rash (OR 0.68, 95% CI 0.56–0.82, FDR adjusted *p* = 0.001) (Figure 2), Stevens-Johnson syndrome (OR 0.18, 95% CI 0.06–0.56, FDR adjusted *p* = 0.027) (Figure 2), and thyroiditis (OR 0.48, 95% CI 0.28–0.81, FDR adjusted *p* = 0.033) (Figure 2).

### 3.4 Association of aspirin treatment with irAEs in different organs

**FIGURE 3:**
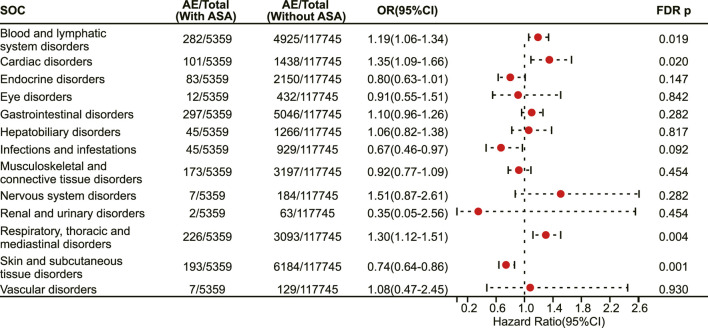
The forest plot showing the association between aspirin use and irAEs from different system organ classes (SOC) among patients receiving immunotherapy. ASA: Aspirin.

Then, we mapped irAEs to their corresponding system organ classes, involving a total of 13 organ systems. Our results demonstrate that aspirin users have a higher risk of developing irAEs in the blood and lymphatic system disorders (OR 1.19, 95% CI 1.06–1.34, FDR adjusted *p* = 0.019) (Figure 3), cardiac disorders (OR 1.35, 95% CI 1.09–1.66, FDR adjusted *p* = 0.020) (Figure 3) and respiratory thoracic and mediastinal disorders (OR 1.30, 95% CI 1.12–1.51, FDR adjusted *p* = 0.004) (Figure 3), while having a lower risk of developing irAEs in the skin and subcutaneous tissue disorders (OR 0.74, 95% CI 0.64–0.86, FDR adjusted *p* = 0.001) (Figure 3).

### 3.5 Association of aspirin use with irAEs among cancer patients treated with PD-1 inhibitors

**FIGURE 4:**
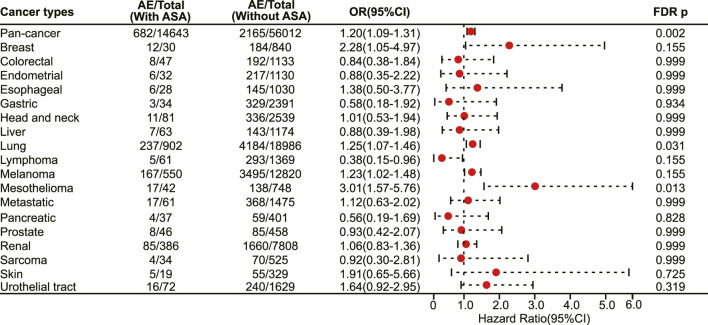
The forest plot showing the association between aspirin use and irAEs across different cancer types among patients using PD-1 inhibitors. ASA: Aspirin.

**FIGURE 5:**
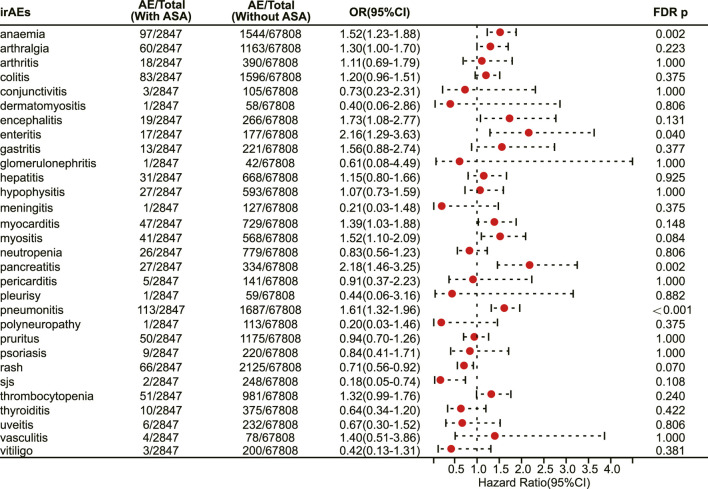
The forest plot showing the association between aspirin use and different irAEs among patients using PD-1 inhibitors. ASA: Aspirin; sjs: Stevens-Johnson syndrome.

We next investigated the association between aspirin use and irAE in patients using different ICIs. In a pan-cancer analysis of patients using PD-1 inhibitors, aspirin use was shown to be associated with a higher risk of irAEs (OR 1.20, 95% CI 1.09–1.31, FDR adjusted *p* = 0.002) (Figure 4). Further analysis revealed that aspirin use was associated with an increased risk of irAEs in lung cancer (OR 1.25, 95% CI 1.07–1.46, FDR adjusted *p* = 0.031) (Figure 4) and mesothelioma (OR 3.01, 95% CI 1.57–5.76, FDR adjusted *p* = 0.013) (Figure 4). In addition, for different adverse reactions, the risk of anaemia (OR 1.52, 95% CI 1.23–1.88, FDR adjusted *p* = 0.002) (Figure 5), enteritis (OR 2.16, 95% CI 1.29–3.63, FDR adjusted *p* = 0.040) (Figure 5), pneumonitis (OR 1.61, 95% CI 1.32–1.96, FDR adjusted *p* < 0.001) and pancreatitis (OR 2.18, 95% CI 1.46–3.25, FDR adjusted *p* = 0.002) (Figure 5) were higher in aspirin users.

### 3.6 Association of aspirin use with irAEs among cancer patients treated with PD-L1 inhibitors

**FIGURE 6:**
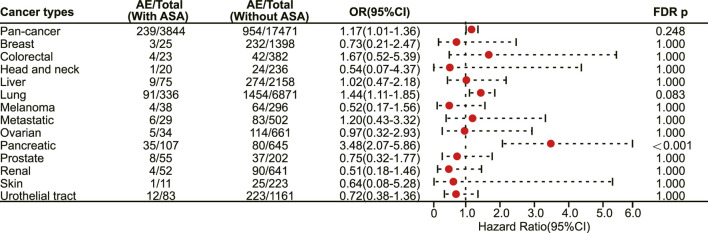
The forest plot showing the association between aspirin use and irAEs across different cancer types among patients using PD-L1 inhibitors. ASA: Aspirin.

**FIGURE 7:**
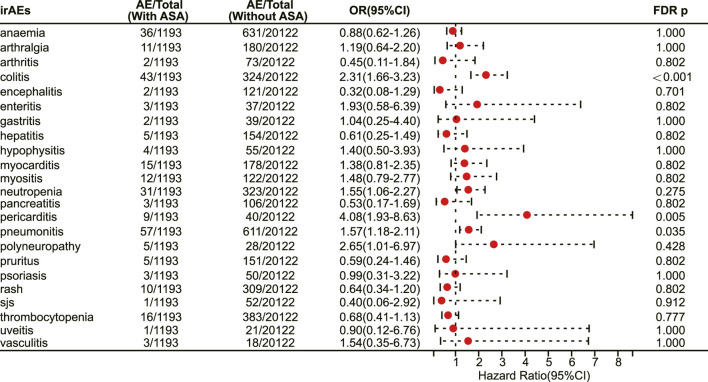
The forest plot showing the association between aspirin use and different irAEs among patients using PD-L1 inhibitors. ASA: Aspirin; sjs: Stevens-Johnson syndrome.

In patients receiving PD-L1 inhibitors, the combination of aspirin demonstrated a tendency to increase adverse reactions in pan-cancer, but there was no statistically significant difference. However, aspirin increased the risk of irAEs in patients with pancreatic cancer (OR 3.48, 95% CI 2.07–5.86, FDR adjusted *p* < 0.001) (Figure 6). In addition, with respect to specific adverse reactions, the risk of colitis (OR 2.31, 95% CI 1.66–3.23, FDR adjusted *p* < 0.001) (Figure 7), pericarditis (OR 4.08, 95% CI 1.93–8.63, FDR adjusted *p* = 0.005) (Figure 7) and pneumonitis (OR 1.57, 95% CI 1.18–2.11, FDR adjusted *p* = 0.035) (Figure 7) were higher in aspirin users.

### 3.7 Association of aspirin use with irAEs among cancer patients treated with CTLA-4 inhibitors

**FIGURE 8:**
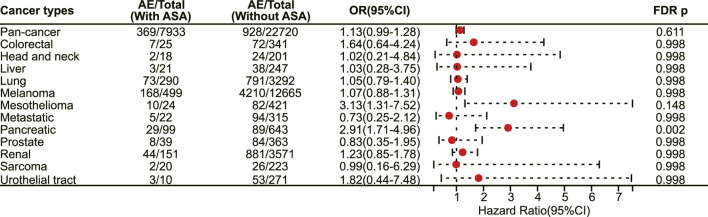
The forest plot showing the association between aspirin use and irAEs across different cancer types among patients using CTLA-4 inhibitors. ASA: Aspirin.

In patients receiving CTLA-4 inhibitors, there is still a trend towards an increased risk of adverse reactions with the use of aspirin, but only with statistical significance in pancreatic cancer (OR 2.91, 95% CI 1.71–4.96, FDR adjusted *p* = 0.002) (Figure 8). No statistical difference was observed among different immune-related adverse events. Finally, subgroup analysis was not performed for patients receiving LAG-3 inhibitors and PD-1/LAG-3 inhibitors due to the small sample size.

### 3.8 Hypothetical molecular mechanisms linking aspirin treatment to the risk of irAEs

**FIGURE 9:**
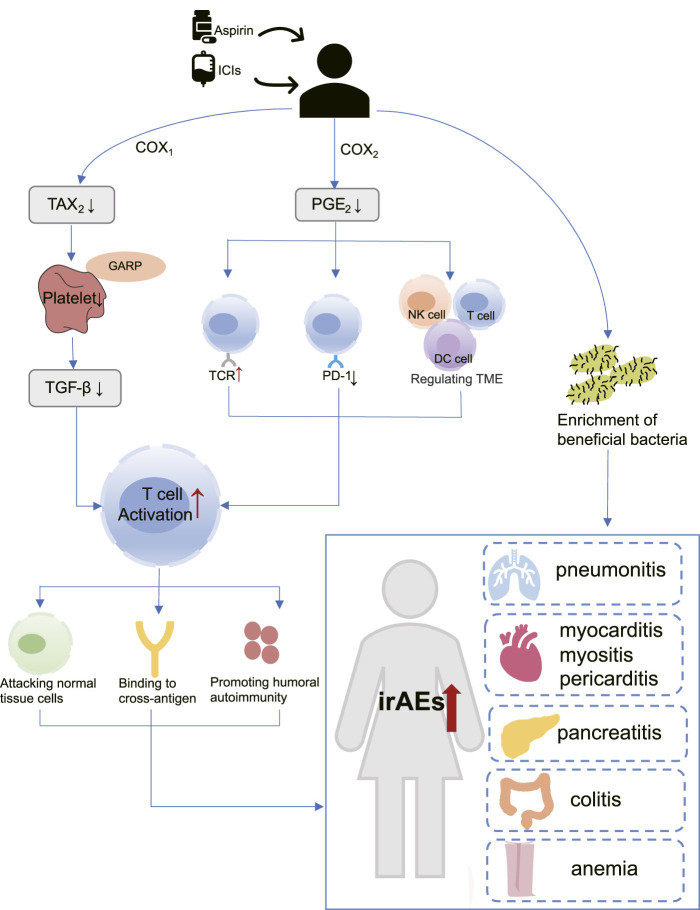
The hypothetical mechanism of increased risk of immune adverse reactions with aspirin use in patients receiving immunotherapy. COX-1: Cyclooxygenase-1; COX-2: Cyclooxygenase-2; DC cell: Dendritic cell; GARP: Glycoprotein A repetitions predominant; ICIs: Immune checkpoint inhibitors; irAEs: Immune-related adverse events; NK cell: Natural killer cell; PGE2: Prostaglandin E2; PD-1: programmed cell death-1; TAX2: Thromboxane2; TGF-β: Transforming growth factor-β; TCR: T-cell receptor; TME: tumor microenvironment.

Until now, the specific mechanisms underlying the association of aspirin with irAEs in cancer patients treated with ICIs remain unknown, but some studies have shown that aspirin plays an important role in immune regulation. Aspirin regulates T cells through COX-1 and COX-2 pathways (Zelenay et al., 2015; Rachidi et al., 2017), and activated T cells may lead to increased irAEs risk (Khan and Gerber, 2020). In addition, the modulation of gut microbiota by aspirin may also mediate the increased risk of irAEs (Chaput et al., 2017) (Figure 9).

## 4 Discussion

By understanding how the immune system interacts with tumor cells, scientists have established new therapies for cancer treatment that have brought noteworthy clinical benefits for cancer patients (Morad et al., 2021). However, many cancer patients have underlying diseases, and the presence of other drugs may affect the immunotherapy. ICIs leverage diverse mechanisms and pathways to harness the immune system’s ability to eradicate tumor cells. Consequently, potential interactions between concomitant medications and ICIs transcend the typical assessment of pharmacodynamic and pharmacokinetic interactions between drugs. Aspirin is currently one of the most widely used basic drugs, Previous studies reported that aspirin use is associated with better outcomes with immunotherapy, However, it is not clear whether aspirin use impacts irAEs. This is one of the first studies to analyze the association between aspirin use with irAEs using FEARS data with innovative and comprehensive benefits. Our study showed that aspirin exposure was associated with an increased risk of irAEs in all enrolled cancer patients treated with ICIs. However, it is important to note that the relationship between aspirin use and irAE risk varies across different tumor types, types of irAEs, and various ICIs. Our research findings highlight these distinctions.

As a well-known non-selective COX inhibitor, aspirin irreversibly acetylates the active sites of COX-1 and COX-2, thereby reducing their enzyme activity (Ornelas et al., 2017). COX-1 mainly mediates the formation of physiological prostaglandins, such as Thromboxane A2 (TXA2), which in turn promotes platelet aggregation (Menter and Bresalier, 2023). Aspirin inactivates COX-1 and prevents the production of TXA2, thus acting as an antiplatelet and preventing thrombosis. More importantly, previous studies have confirmed the role of platelets in promoting tumor growth and metastasis (Lichtenberger and Vijayan, 2019). Rachidi et al. (2017) found that a protein called Glycoprotein A repetitions predominant (GARP) exists on the surface of platelets, which traps and activates Transforming growth factor-β (TGF-β). TGF-β is an immunomodulatory molecule that suppresses CD4 and CD8 T cells, allowing tumors to evade the immune system. Riesenberg et al. (2019) confirmed through a mouse model that the antiplatelet effect of aspirin can inhibit TGF-β signaling, thereby enhancing T cell function, and synergistically exerting anti-tumor effects with PD-1 blocker.

COX-2 is an inducer of enzymes that promote the synthesis of inflammatory prostaglandins, such as Prostaglandin E2 (PGE2), which can cause inflammation (Jin et al., 2023). Interestingly, PGE2 has been shown to regulate the function of various immune cells within the tumor microenvironment (TME), including myeloid-derived suppressor cells (MDSCs), dendritic (DC) cells, natural killer (NK) cells, CD4 and CD8 T cells, resulting in immune evasion (Zelenay et al., 2015; Böttcher et al., 2018; Bonavita et al., 2020). Moreover, PGE2 is capable of upregulating PD-L1 expression (Goto et al., 2020) and inhibit T cell receptor activation (Newick et al., 2016). The above study suggests that aspirin may exert immunomodulatory effects and enhance T cell activation by inhibiting COX2/PGE2 pathway (Wei et al., 2022; Jin et al., 2023).

Aspirin has been found to aid ICIs in breaking immune tolerance and amplifying the immune response (Zelenay et al., 2015). Unfortunately, it is important to note that immune activation is not limited to tumor-specific responses. Some researchers have proposed that activated effector T cells also attack normal non-tumor tissues while increasing their anti-tumor activity (Khan and Gerber, 2020; Ronen et al., 2022). T-cell receptor (TCR) sequencing studies have provided evidence to support this theory (Porciello et al., 2022; Sanromán Á et al., 2023). In patients treated with Ipiliumumab, researchers detected greater CD4 and CD8 T cell diversity in irAEs patients compared with those who did not experience significant adverse reactions (Oh et al., 2017). A recent work from Luoma and others has demonstrated the presence of a large number of CD8 T cells with high cytotoxicity and proliferation ability in the colon of patients with colitis, and these CD8 T cells are mostly from tissue-resident populations (Luoma et al., 2020). Together, these studies support that irAEs may be caused by the mobilization of a large number of T cells (Ramos-Casals et al., 2020). Other studies have shown that the presence of cross-antigens can also influence T-cell responses. In a study by Berner et al., 73 patients with NSCLC who received anti-PD-1 treatment were included, and nine common T-cell antigens were identified between tumor tissues and skin. This indicates that ICIs target both non-small-cell lung cancer (NSCLC) cancer and skin, leading to immune-related dermal toxicity while treating tumors (Berner et al., 2019). On the other hand, self-antigens from dying cells are captured by antigen-presenting cells (APCs) during tumor cell killing. These APCs then migrate to lymph nodes and activate more reactive T and B cells, These novel T cell clones may initiate a distinct immuno-editing wave, leading to adverse reactions (Yost et al., 2019; Baumjohann and Brossart, 2021).

Multiple clinical studies have investigated the potential of aspirin in enhancing the immune response in immunotherapy. Cortellini et al. (2020) reported that concurrent use of aspirin can improve overall response rate (ORR) among patients with solid tumors receiving PD-1/PD-L1 checkpoint inhibitors. Another study have highlighted that aspirin can prolong overall survival (OS) (Kostine et al., 2021). Furthermore, a meta-analysis suggested a significant intensification in progression-free survival (PFS) with concurrent use of aspirin and ICIs (Zhang et al., 2021). The above statements have demonstrated the synergistic effect of aspirin in ICIs. Therefore, aspirin may have underestimated immunomodulatory effects can amplify immune activation induced by ICIs. However, coins always have two sides. Over-activated T cells lack tumor specificity, so we have to consider the impact of aspirin on irAEs. We propose that aspirin may enhances T cell activation through inhibition of PGE2 and platelets, contributing to the increased irAEs.

Moreover, it has been shown that microbiota composition was a key factor in maintaining immune homeostasis, and may affect the occurrence of irAEs (Dora et al., 2023). Chaput et al. (2017) demonstrated that protective bacteria in the gut led to positive outcomes for patients who receive ipilimumab therapy, but also with a higher incidence of ipilimumab-induced colitis. Mouse models have shown that aspirin modulates the gut microbiota by enrichment of probiotics (Zhao et al., 2020; Brennan et al., 2021). This may also be one of the reasons why aspirin is associated with an increased risk of irAEs occurring (Figure 9).

Our research has uncovered a connection between the use of aspirin and an increased susceptibility to irAEs in pan-cancer patients. Delving deeper into our findings, we have identified a notably increased risk of irAEs among patients afflicted with specific cancer types, including lung cancer, mesothelioma, and pancreatic cancer. Conversely, a perplexing reduction in irAE risk has emerged in lymphoma patients. Remarkably, these observations constitute a novel contribution to the field, as they have not been previously documented in existing literature.

In stark contrast to prior retrospective studies, our comprehensive analysis has demonstrated robust statistical significance in support of these findings (Gandhi et al., 2020; Sieber et al., 2022). We posit that aspirin’s influence on the occurrence of irAEs may be mediated through the COX pathway, thereby shedding light on a potential mechanistic explanation. Furthermore, the intriguing divergence observed within the lymphoma subgroup warrants further investigation. While our data show a diminished risk of irAEs in lymphoma patients, it is essential to acknowledge that this subgroup comprises a relatively small sample size, constituting only 1.5% of the overall study population. It is conceivable that this statistical anomaly may be attributed to the limited representation of lymphoma cases, or it may signify the existence of hitherto undiscovered mechanisms that demand further exploration and scrutiny. In addition, aspirin use, prescribing status, or combination of aspirin with these conditions. These circumstances will also have an impact on our results (Colard-Thomas et al., 2023).

Our in-depth analysis revealed a significant association between the use of aspirin and a range of irAEs. Specifically, we observed that aspirin use markedly increased the risk of patients experiencing irAEs such as pneumonia, myocarditis, myositis, pericarditis, pancreatitis, colitis, and anemia. In contrast, the risk of irAEs related to conditions like rash, Stevens-Johnson syndrome, and thyroiditis was notably reduced. To further support our conclusions, we conducted a comprehensive review of previously published articles, seeking evidence that aligns with the associations we identified. Prior studies may not have fully considered the relationship between aspirin and irAEs or may not have detected these associations due to differences in research methodologies. Nonetheless, our study fills this knowledge gap and provides healthcare professionals with a more comprehensive understanding of aspirin’s role in irAE risk.

In summary, these findings underscore the need for heightened vigilance among clinicians when treating patients with immunotherapy, especially in cases related to irAEs affecting organs or systems such as the gastrointestinal tract, lungs, pancreas, heart, and anemia. However, it is also essential to consider an additional factor, namely, the widespread use of aspirin in cardiovascular disease treatment (Byrne and Colleran, 2020), where a patient’s history of cardiovascular conditions may be one of the factors contributing to the heightened risk of irAEs (Yousif et al., 2023). Therefore, a comprehensive assessment of the patient’s overall health and treatment needs is crucial.

Despite some limitations in our study and a lack of supporting mechanistic research, our research still provides valuable pharmacological guidance to the greatest extent possible. For example, when using aspirin in patients receiving PD-1 inhibitors, it is advisable to pay closer attention to indicators related to anemia, enteritis, pneumonia, and pancreatitis. Similarly, for patients undergoing PD-L1 inhibitor treatment, increased attention should be directed towards indicators associated with colitis, pericarditis, and pneumonia. Furthermore, in patients receiving CTLA-4 inhibitors, no association has been observed between aspirin and irAEs, although further research is needed to confirm this, in order to offer clinicians more precise treatment guidelines.

Overall, our study highlights the potential risks associated with aspirin use in patients receiving immunotherapy, particularly with regards to irAEs. These findings could inform clinical decision-making and improve patient safety.

## 5 Study limitations

The FAERS database, as a voluntary, passive, and non-mandatory reporting system, faces inherent challenges. These include incompleteness, inaccuracy, inconsistency, and delay in reporting adverse events. These limitations stem from various factors, primarily the lack of detailed patient characteristics, drug exposure information, and outcome details, such as the dose and duration of aspirin use, as well as whether patients received other treatment regimens and the sequence of medication. These factors may influence the associations observed and the study outcomes. Therefore, it is essential to carefully consider these limitations, particularly when interpreting the research results.

Furthermore, our analysis is influenced by the uneven distribution of cases within the database, with a higher number of lung cancer patients but significantly fewer patients with other cancer types. This non-uniform case distribution may introduce bias and restrict the generalizability and applicability of our study findings.

To overcome these limitations and provide more robust insights, further prospective clinical studies are urgently needed. Additionally, the mechanisms underlying the association between aspirin use and irAEs remain unclear, underscoring the need for fundamental research to address these uncertainties and advance our understanding of immunotherapy.

## 6 Conclusion

This study has revealed a significant association between aspirin usage and irAEs in cancer patients undergoing ICIs. It is important to note that this association exhibits variations depending on the specific cancer type, the nature of adverse events, and the specific type of ICIs being utilized. These findings underscore the importance of assessing the effect of baseline drugs, including aspirin, on the safety and efficacy of ICIs in tumor treatment, and tailoring treatment plans accordingly on an individual basis.

## Data Availability

The original contributions presented in the study are included in the article/Supplementary Material, further inquiries can be directed to the corresponding author.
